# Endoscopic sinus surgery (ESS) to change quality of life for adults with recurrent rhinosinusitis: study protocol for a randomized controlled trial

**DOI:** 10.1186/s13063-021-05576-z

**Published:** 2021-09-08

**Authors:** Heidi M. Kaski, Antti Alakärppä, Ulla Lantto, Aleksi Laajala, Paulus Tokola, Tomi Penna, Pasi Ohtonen, Olli-Pekka Alho

**Affiliations:** 1 Department of Otorhinolaryngology and Head and Neck Surgery, Oulu University Hospital, P.O. Box 5000, FIN-90014 Oulu, Finland; 2 PEDEGO Research Unit, University of Oulu, Oulu, Finland; 3 Division of Operative Care, Oulu University Hospital, Oulu, Finland; 4 Medical Research Center Oulu, Oulu, Finland

**Keywords:** Rhinosinusitis, Recurrent rhinosinusitis, Endoscopic sinus surgery, Quality of life, Treatment, Randomized controlled trial

## Abstract

**Background:**

Endoscopic sinus surgery (ESS) has been used for decades to treat recurrent acute rhinosinusitis episodes (RARS) in adults. RARS results in infectious symptoms, antibiotic courses, sick leaves, and impaired quality of life. Theoretically, the ESS procedure, through improving the drainage of the paranasal sinuses, decreases the symptoms and enhances the quality of life of the RARS patients. Whether this is true has not been reported in a randomized trial yet.

**Methods:**

We conduct a single-center, non-blinded, randomized, 6-month, parallel group superiority clinical study including 80 adult participants referred to surgical treatment for RARS. The participants will either have ESS or conservative medical treatment (control group). The primary outcome will be the difference between the mean disease-specific Sinonasal Outcome Test 22 (quality of life questionnaire) change scores (from baseline to 6 months) of ESS and control group.

**Discussion:**

This study will add significant new information to the effect and harms of ESS procedure in the treatment of adults with RARS.

**Trial registration:**

ClinicalTrials.govNCT04241016. Registered on 17 January 2020

**Supplementary Information:**

The online version contains supplementary material available at 10.1186/s13063-021-05576-z.

## Administrative information

The order of the items has been modified to group similar items (see http://www.equator-network.org/reporting-guidelines/spirit-2013-statement-defining-standard-protocol-items-for-clinical-trials/).

**Table:** 

Title {1}	Endoscopic sinus surgery (ESS) to improve quality of life for adults with recurrent rhinosinusitis
Trial registration {2a and 2b}.	ClinicalTrials.gov registration number NCT04241016
Protocol version {3}	25.8.2021 version number 7
Funding {4}	Governmental research grant No external funding
Author details {5a}	Kaski H, MD, doctoral student Alakärppä A, MD, PhD, specialist in Otorhinolaryngology Lantto U, MD, PhD, specialist in Otorhinolaryngology Laajala A, MD, specialist in Otorhinolaryngology Tokola P, MD, specialist in Otorhinolaryngology Penna T, MD, specialist in Otorhinolaryngology Ohtonen P, MSc, statistician Alho O-P, MD, PhD, professor of Otorhinolaryngology Department of Otorhinolaryngology and Head and Neck surgery, Oulu University Hospital, Oulu, Finland PEDEGO research unit, University of Oulu, Finland Medical Research Center Oulu, Oulu, Finland Division of Operative Care, Oulu University Hospital, Oulu, Finland
Name and contact information for the trial sponsor {5b}	Trial Sponsor: Oulu University Hospital Sponsor’s Reference: Y-0679480-9 Contact name: Ms. Minna Mäkiniemi Address: P O Box 10, FIN-90029 Oulu University Hospital, Finland Telephone: + 358 8 315 2011 Email: minna.makiniemi@ppshp.fi
Role of sponsor {5c}	The funders have no role in the design, data collection and analysis, decision to publish or the preparation of the manuscript

## Introduction

### Background and rationale {6a}

Endoscopic sinus surgery (ESS) is among the most common surgical procedures in the field of ear, nose, and throat diseases. In Finland, 60 ESS procedures per 100,000 adult residents are performed on average per year [1]. Patients with recurrent acute rhinosinusitis (RARS) form a substantial proportion of those undergoing this procedure. In our population-based cohort study, where we examined the quality of life (QoL) benefits of primary sinonasal surgery, some 40% of the patients suffered from RARS [2].

Patients with RARS face disturbing symptoms, significant financial burden in the form of recurrent absences from work, health care visits and medical treatment costs [3, 4], and social harms. That is, their QoL is impaired. RARS has been observed to lower both the disease-specific and generic QoL, even to an extent similar to chronic rhinosinusitis [2, 5].

Current treatment recommendations for RARS include intranasal corticosteroids, antibiotics, and, in case conservative treatment fails, ESS. According to the literature, surgery becomes an option after 4 episodes per year [2, 3, 5, 6]. ESS contains surgical opening of the ethmoidal sinuses and enlargement of the maxillary sinus ostium. The procedure is done under local or general anesthesia as a day surgery. Theoretically, with improved drainage of the ethmoidal and maxillary sinuses the accumulation of bacterial fluid in the sinuses is diminished. This is thought to decrease the number and severity of the recurrent episodes and increase patients’ QoL. The possible harms related to ESS include postoperative pain, hemorrhage, infections, and rarely orbital or intracranial complications and even death [7–9].

Observational studies have indicated that ESS encompasses several benefits among patients with RARS. It has been reported to decrease the numbers of rhinosinusitis episodes, antibiotic courses, and missed workdays [3, 4]. Moreover, several observational studies claim it enhances both the disease-specific [2, 4–6, 10, 11] and general QoL [2, 5] in adults with RARS. According to these studies the harms related to ESS are mild in terms of the frequency and severity of the complications.

At present, we are not aware of any randomized controlled trial that would have explored the change of either researcher-measured or patient-related outcome measures after ESS among RARS patients. With this trial, we aim at exploring the beneficial and harmful effects of ESS on QoL, patients’ health and health care utilization as compared to conservative treatment in these patients.

### Objectives {7}

Our primary objective is to compare the change in disease-specific QoL between ESS and control group, but we will also compare the alterations in general QoL and the number of rhinosinusitis episodes, symptomatic days, antibiotic courses, visits to medical care, and days lost from work or study. We will also monitor the adverse effects in the surgical group. We hypothesize that ESS procedure is superior to conservative treatment in improving the disease-specific quality of life without significant risks among adults suffering from RARS.

### Trial design {8}

This study is designed as a single-center randomized, controlled, parallel-group, open, superiority trial with a follow-up period of 6 months. Participants will be assigned to two groups using block randomization with a 1:1 allocation ratio.

## Methods: participants, interventions, and outcomes

### Study setting {9}

The study is conducted at Oulu University Hospital in Finland. We will recruit patients referred to this hospital’s outpatient ear, nose, and throat clinic. All cases come from a population of 405,000 inhabitants in Northern Finland. This is the secondary care area of the Oulu University Hospital and is comprised of 29 municipalities that maintain one primary health center each and one university hospital collectively (Oulu University Hospital). Practically, all sinonasal surgeries in the area are done in this hospital. The health care system in Finland is based on a general health insurance scheme and provides equal access to medical services for all citizens. All patients must first present in primary care before referral to secondary or tertiary care.

### Eligibility criteria {10}

#### Inclusion criteria

The inclusion criteria are as follows:

- At least 3 acute rhinosinusitis episodes in 6 months or 4 episodes in 12 months.
- These episodes must last less than 4 weeks and be diagnosed and treated as acute rhinosinusitis by a physician.
- During an episode the participants must have typical acute sinusitis symptoms consisting of nasal discharge, nasal congestion, hyposmia, and facial pressure or pain and the episodes have to be severe enough for the participant to seek medical help and for daily life to be significantly disturbed.
- Participants must have failed conservative therapy of 3 months adhering to the Finnish treatment guidelines [12] and the Canadian clinical practice guide [13].

If conservative treatment has been inadequate at the time of referral, the participants may first receive conservative treatment and then be again considered for enrolment if other criteria are fulfilled. These criteria for RARS follow those presented by The International Consensus Statement on Allergy and Rhinology [14].

#### Exclusion criteria

Potential participants have to be excluded in the following cases:

- Age under 18 years
- Immunodeficiency or immunosuppression
- Pregnancy
- Previous illness making same-day surgery unfeasible
- Ongoing antibiotic treatment for other reasons
- Primary complaint of nasal septal deviation and
- Chronic rhinosinusitis with or without nasal polyposis

We define chronic rhinosinusitis as nasal discharge, nasal congestion, hyposmia, and facial pressure or pain lasting at least 12 weeks with endoscopical or radiological (paranasal computed tomography (CT) or cone-beam computed tomography (CBCT) findings matching the diagnosis. In assessing CT/CBCT and endoscopic findings, we use Lund-Mackay and Lund-Kennedy scoring system, respectively [15, 16]. Any pathological endoscopic finding in the middle meatal area or Lund-Mackay score over 4 points are considered as chronic rhinosinusitis. Immunodeficiencies are asked from the patients and tested from blood samples. Patients with neutropenia (neutrophil count < 1.5 × 10^9^/l), lymphocytopenia (lymphocyte count < 1.2 × 10^9^/l), or immunoglobulin G or A deficiency (serum IgG < 6.77 g/l, IgA < 0.88 g/l for men and < 0.52 g/l for women) are excluded. Courses of oral corticosteroids for rhinosinusitis are allowed but patients with other immunosuppressive medication are excluded.

### Who will take informed consent? {26a}

The participants will be recruited from consecutive adult patients referred to the ear, nose, and throat outpatient department at the Oulu University Hospital (Oulu, Finland) because of rhinosinusitis problems. Recruitment is done by five research members from the department of ear, nose, and throat. Based on the inclusion and exclusion criteria, patients will be screened for study participation. The participants are interviewed, and written evidence of previous acute rhinosinusitis episodes is looked for from referral letters and patient files. Other clinical criteria are asked from the participants, and blood samples, nasal endoscopy, and CT or CBCT scans from the paranasal area are taken to rule out immunodeficiencies and chronic rhinosinusitis, respectively. Those who fulfill the eligibility criteria and express an interest in participating in the study will be given a verbal explanation of the study details and of the written consent and any questions regarding the study will be answered. Then, each participant will have sufficient time to decide whether to participate in this study. For those willing to participate, written consent will be obtained.

### Additional consent provisions for collection and use of participant data and biological specimens {26b}

On the consent form, participants will be asked if they agree to the use of their data, should they choose to withdraw from the trial. This trial does not involve collecting biological specimens for storage.

## Interventions

### Explanation for the choice of comparators {6b}

The surgical treatment, ESS, is compared to conservative treatment of RARS, which current guidelines recommend as the primary mode of treatment [12, 13]. Using usual care as a comparator ensures that the effect of ESS is not over- or underestimated and that the patients are more willing to participate.

### Intervention description {11a}

ESS will be performed as day-surgery under local or general anesthesia. Endoscopic equipment for ESS from Olympus Europe (Hamburg, Germany) and Karl Storz (Tuttlingen, Germany) and powered surgical instruments manufactured by Medtronic (Jacksonville, Florida) will be used. Preoperative CT/CBCT scans will be routinely utilized in the operation, but intraoperative surgical navigation in only cases with exceptionally complicated anatomy. The residents and specialists of ear, nose, and throat diseases at the Oulu University Hospital will perform the operations. Topical anesthesia of the nose (lidocaine-adrenalin gauzes and infiltration, and topical cocaine hydrochloride) will be used to control bleeding and thus to achieve better visibility regardless of the use of general anesthesia. The surgical procedure will follow the techniques presented by Messerklinger and Stammberger [17] with uncinectomy, conservative middle meatal antrostomy, and appropriate ethmoidectomy. Ethmoid cells with pathological appearance on CT/CBCT will be opened, but since we exclude patients with major radiological findings, we anticipate that minimal anterior ethmoidectomy will be done to most participants. Septoplasty may be performed according to evaluation of the surgeon for improved access. Postoperative treatment consists of daily nasal douching and topical corticosteroids for 2 weeks, pain medication, and at least one postoperative control visit including endoscopy 2 weeks after the operation.

Conservative treatment in the control group consist of daily nasal corticosteroids, nasal douching, courses of antibiotics during acute episodes, and other oral and/or nasal allergy medication, if appropriate. The details of individual participant’s treatment will be decided according to the attending clinician’s judgment and Finnish and Canadian clinical guidelines [12, 13].

### Criteria for discontinuing or modifying allocated interventions {11b}

The assigned study intervention may be discontinued for withdrawal of participant consent mainly. The participant may refuse to have the assigned surgery or discontinue the conservative treatment, which serves as control. The group assigned surgery will be operated on within 2 weeks after enrolment, so by looking after proper criteria for study entry, we will minimize the risk of refusal. Similarly, the group assigned conservative treatment will eventually be operated on after 6 months follow-up when this trial is over. The waiting time here, which is within the normal limits for our hospital, decrease the risk of participants seeking the operation elsewhere. For these reasons, no standard criteria for discontinuations are designed. In case of participants discontinuing their assigned intervention, we still aim at collecting the outcome data as planned to prevent missing data.

### Strategies to improve adherence to interventions {11c}

As suggested by Little et al. [18], the following suggestions for limiting missing data in the design and conduct of clinical trials are used. The target population of RARS patients is prior to the study not adequately served by treatments and hence has an incentive to remain in the study. The control group is allowed a flexible treatment regimen that accommodates individual differences in efficacy and side effects in order to reduce the dropout rate because of lack of efficacy or tolerability. The follow-up period for the primary outcome will be relatively short. We select investigators who have a good track record with respect to enrolling and following participants and collecting complete data in previous trials. We set 10% as an acceptable target rate for missing data concerning the primary outcome and will monitor the progress of the trial with respect to this target. We limit the burden and inconvenience of data collection on the participants and make the study experience as positive as possible. We train investigators and study staff that keeping participants in the trial until the end is important, regardless of whether they continue to receive the assigned treatment. We also convey this information to study participants. We collect information from participants regarding the likelihood that they will drop out and use this information to attempt to reduce the incidence of dropout. We keep contact information for participants up to date. One of the study nurses will call the participants at three months follow-up to remind them of data collection as well as their treatment plan.

### Relevant concomitant care permitted or prohibited during the trial {11d}

During the follow-up period, the participants in both groups are allowed standard treatment of rhinosinusitis symptoms, including antibiotics, topical, and systemic medications. Typically, these include nasal lavages with normal saline solution, topical nasal or per oral steroids (e.g., daily doses of topical beclomethasone 100 μg, budesonide 100–200 μg, fluticasone 100 μg, mometasone 100 μg, or per oral prednisolone 5–10 mg) and per oral antihistamines with or without sympathomimetic agent (e.g., loratadine 10 mg, ebastine 10 mg, desloratadine 2.5–5 mg (+ pseudoephedrine 120 mg), cetirizine 5–10 mg (+ pseudoephedrine 120 mg), acrivastine 8 mg + pseudoephedrine 80 mg). These medications are used during the acute episodes or continuously if the symptoms are chronic or recur frequently.

### Provisions for post-trial care {30}

There is no anticipated harm and compensation related specifically to trial participation. The assigned treatments are ordinary, and the participants are covered by the regular malpractice insurance.

### Outcomes {12}

We will collect background information with a questionnaire. This includes data on age, gender, weight and height, smoking status, education, illnesses, performance status, allergic rhinitis, immunotherapy for allergic diseases, domestic animals, exposures to moldy buildings and irritants, number of rhinosinusitis episodes per year, presence of respiratory symptoms and medications used during the prior month, and number of medical visits during the prior month. Participants undergo clinical examination, nasal endoscopy, and CT/CBCT of the paranasal area. Sensitivity to the most common aeroallergens is explored using either skin prick testing or serum immunoglobulin E assay.

The participants are given a study notebook, which includes information about the study and written instructions for their general practitioners of recording the date, clinical findings, diagnoses, and treatments in case participants seek medical care for respiratory symptoms. Notebook also contains anchor questions about the overall disturbance caused by the respiratory symptoms using a using a 7-point global rating, reasons for seeking medical care initially, and participant’s expectations of the treatment. Participants keep symptom diary and grade (from 0 = no to 3 = very severe) daily the following symptoms: nasal obstruction, nasal discharge, facial pain/pressure, nasal pain, nasal bleeding, fever, and absence from work or study.

To record quality of life, we use the disease specific Sinonasal Outcome Test – 22 (SNOT-22) and general Research and Development 36-item Health Survey (RAND-36) questionnaires. SNOT-22 is developed specifically for measuring the impact of chronic rhinosinusitis on QoL [19, 20], and it records patient-reported symptoms and disability from the previous two weeks. SNOT-22 is regarded as a reliable, valid, and responsive disease-specific instrument [20–22]. SNOT-22 produces a summary score between zero and 110, with higher values indicating poorer disease-specific QoL. RAND-36 is a short-form health survey developed as a tool for outcome measurement in the Medical Outcomes Study [23]. RAND-36 is divided into eight domains, which measure generic health-related QoL. The domains are physical functioning, role limitations due to physical health or emotional problems, energy/fatigue, emotional well-being, social functioning, pain, and general health. The instrument’s scoring algorithm produces eight individual values between 0 and 100 for each domain, with higher scores indicating better QoL. We used the Finnish translations of both instruments, which have been translated, culturally adapted, and validated [24, 25].

## Primary outcome

The primary outcome is as follows: difference between the mean disease-specific SNOT-22 change scores (from baseline to 6 months) of ESS and control group. Distributions of different SNOT-22 domain scores are reported in ESS and control group as well.

## Secondary outcomes

1. Difference between the mean generic RAND-36 domains change scores (from baseline to 6 months) of ESS and control group.
2. Difference in proportions of participants benefiting clinically significantly from the treatment in ESS and control group (minimum important change in SNOT-22 score) at 6 months.
3. Difference in the numbers of rhinosinusitis episodes, medical visits, antimicrobial treatments, and days lost from work or studies between the ESS and control groups during the follow-up.
4. Difference in the numbers of days with nasal obstruction, nasal discharge, facial pain or pressure, nasal pain, nasal bleeding, and fever between the ESS and control groups during the follow-up.
5. Frequency of postoperative synechia formations, postoperative infections, and orbital and intracranial complications in the ESS group during the follow-up.

Although we think that health care utilization is an important issue in paranasal surgery, we still feel that patient’s perspective is as important. As RARS mainly lowers quality of life, an effective treatment should primarily enhance it without substantial harms. Therefore, we chose the quality of life change after ESS as our primary outcome and recorded the clinically relevant possible harms and health utilization change as secondary outcomes.

### Participant timeline {13}

Participants in the intervention (ESS) group will be operated as soon as practically possible which we estimate to be within 2 weeks of assignment. The participants in the control group will be placed on a waiting list for ESS to undergo surgery after 5 to 6 months, which is the usual operational delay for elective surgery in our clinics. For the control group, follow-up will finish before the participants are operated.

The enrolment, interventions, assessments, and study visits of our trial are presented in Table 1.

**Table 1:**
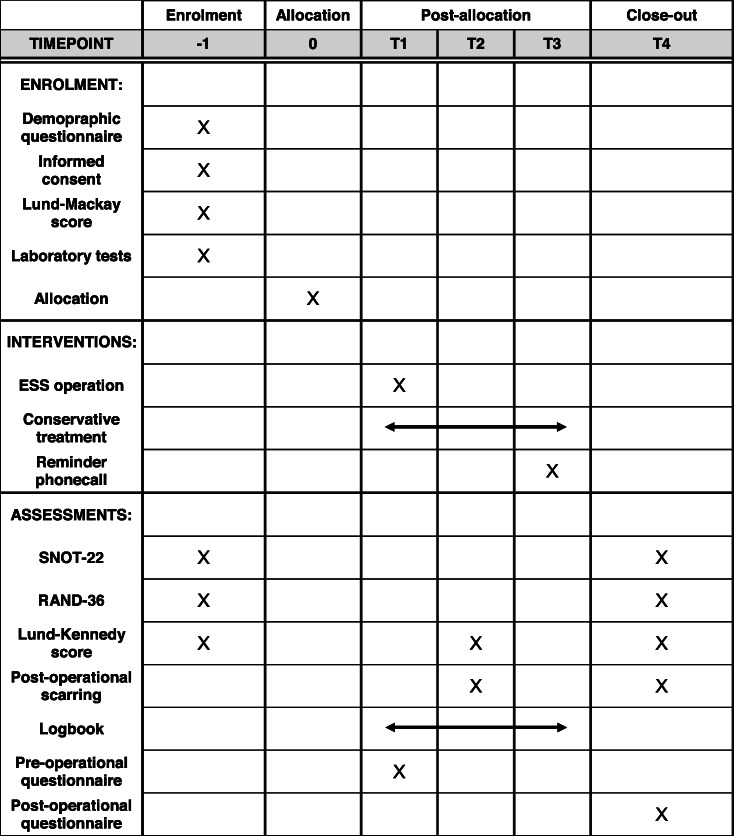
The schedule of enrolment, interventions, and assessments. T1 = within 2 weeks of allocation, T2 = 2 weeks after endoscopic sinus surgery, T3 = 3 months follow-up, T4 = 5–7 months. The study logbook is filled at home continuously between allocation and the end of follow-up. ESS, endoscopic sinus surgery; SNOT-22, Sinonasal Outcome Test 22; RAND-36, Research and Development 36-item Health Survey

### Sample size {14}

Based on our earlier cohort study on the effect of endoscopic sinus surgery on recurrent sinusitis, we anticipate that the SNOT-22 score at the end of 6 months follow-up is 30 (SD 19) in the control group and 20 in the surgical group [2]. We estimate that 78 participants need to be enrolled in the study for it to have statistical power of 80% to detect the above-mentioned difference, which is also about the same as the minimum important change for the SNOT-22 questionnaire described in the literature. We consider a 2-sided *p* value of 0.05 to be significant. A two-step method proposed by Borm et al. is used for this sample size estimation [26]. First, the sample size is calculated as if a *t* test on the follow-up scores is carried out. Second, the numbers of subjects required for the study is multiplied by 1-*ρ*^2^, where *ρ* is the correlation between baseline and follow-up scores, again derived from our earlier cohort data (Pearson correlation 0.577). To compensate any loss to follow-up, we decide to enroll 80 participants.

### Recruitment {15}

Our trial team includes five research members from the department of ear, nose, and throat who will be in charge of the recruitment process. We expect the contested sample size of 80 participants to be recruited by 2024. The research team follow-ups the actualized recruitment rate regularly during the bimonthly meetings. No financial or non-financial incentives are provided to trial investigators or participants for enrolment.

## Assignment of interventions: allocation

### Sequence generation {16a}

Eligible participants will be randomly assigned to ESS or control group (1:1 ratio) as per a computer-generated randomization schedule using permuted blocks of random sizes. The block sizes will not be disclosed, to ensure concealment.

### Concealment mechanism {16b}

The allocation sequence will be concealed from the investigators enrolling participants by putting the assigned treatments in sequentially numbered, opaque, sealed envelopes.

### Implementation of allocation {16c}

As suggested in the SPIRIT 2013 Statement, we strive for complete separation of the individuals involved in the steps before enrolment from those involved in the implementation of study group assignments [27]. A biostatistician, not involved in assignment or care of the trial participants, generates the randomization sequence with a computerized random number generator. A research assistant, not involved in any other aspect of this study, will place the assigned treatments in the sealed envelopes. Participants who fulfill the inclusion criteria will be recruited by the ear, nose, and throat specialists involved in the trial. Allocation envelopes will be opened sequentially only after an eligible participant has been found, informed consent obtained, and after a study nurse has attached a self-fastening slip of paper containing the participant’s name to the official study files.

## Assignment of interventions: blinding

### Who will be blinded {17a}

Blinding of the participants and care providers is not feasible because of the obvious differences between the interventions. As patients are usually referred to our clinics with the expectation of surgical intervention, sham operations would make recruiting difficult.

### Procedure for unblinding if needed {17b}

Not applicable.

## Methods: data collection, management, and analysis

### Plans for assessment and collection of outcome, baseline, and other data {18a}

Study candidates are evaluated at the ear, nose, and throat outpatient department of the Oulu University Hospital by the trial investigators. Data from interviews, referral letters, patient files, blood samples, and nasoendoscopical and radiological examinations are used to evaluate whether these candidates fulfill the eligibility criteria. The endoscopic and radiologic findings are scored according to suggested guidelines [15, 16]: endoscopic findings are assessed with Lund-Kennedy scores and radiological findings with Lund-Mackay stages. Baseline information is gathered with a questionnaire and allergy testing. Allergy testing includes serum immunoglobulin E and an immunofluorometric assay of the most common allergen-specific IgEs in Finland (*Cladosporium herbarum*, *Dermatophagoides pteronyssinus*; horse, cat and dog dandruff; birch, mugwort and timothy pollen). Outcome data is collected with study notebook, which includes anchor questions, and data on medical visits, sick leaves, and various symptoms during the follow-up. The SNOT-22 and RAND-36 questionnaires filled at baseline and at the end of follow-up. The Finnish versions of the quality of life instruments have been found to be reliable, valid, and responsive [24, 25]. Possible ESS complications are collected at the postoperative visits and at the end of the follow-up from the notebook and patient files. For the baseline questionnaire and study notebook, see Additional files 1 and 2, respectively. For the radiological and endoscopical assessment forms, see Additional file 3.

### Plans to promote participant retention and complete follow-up {18b}

To promote retention, we stress the importance of this trial to RARS patients and physicians treating them, provide the participants feedback from the results of the medical examinations made, and call them to remind to fill the study logbook and about the follow-up visit. We will take care of that all randomized participants will fill the quality of life questionnaires at baseline and at the end of follow-up in case they have not done so in the study logbook. For those participants who do not show up to the follow-up visit, we shall try to get answers to these questionnaires by phone to avoid missing outcome data. All cases are analyzed in an intention to treat principle. Participants may withdraw from the study at any point they want to. The reason for withdrawal is recorded if the participant allows.

### Data management {19}

The demographic data and that collected from the baseline questionnaires, clinical, radiological and laboratory examinations, patient files, and study notebooks are collected using paper case report forms. One of the authors (H.K.) will create a coded SPSS file from these forms, which is then commented and revised by the whole research group. She will then enter the data into digital form with single-data entry method. To verify the accuracy of data entry and coding, the following methods are implemented: verification that the data are in the proper format or within an expected range of values and independent source document verification of a random subset of data.

### Confidentiality {27}

Participants’ confidentiality is secured by (1) the creation of coded, depersonalized data where the participant’s identifying information is replaced by an unrelated sequence of characters, (2) secure maintenance of the data and the linking code in separate locations using encrypted digital files within password protected folders and storage media, (3) keeping paper data in a locked cupboard that only study personnel will be able to access in an area with restricted access, and (4) limiting access to the minimum number of individuals necessary for quality control, audit, and analysis (H.K., P.O., O.-P.A.). Participant files will be stored for 2 years after the completion of the study. The data are not transmitted elsewhere.

### Plans for collection, laboratory evaluation, and storage of biological specimens for genetic or molecular analysis in this trial/future use {33}

This trial does not involve collecting, laboratory evaluation, or storage of biological specimens for genetic or molecular analysis.

## Statistical methods

### Statistical methods for primary and secondary outcomes {20a}

To describe the data the following methods will be used. Data for normal distribution will be presented as mean and standard deviation (SD). Variables for skewed distributions will be described as median and interquartile range. Categorical variables will be expressed as frequencies with percentages.

The primary and secondary outcomes, measures and planned statistical analyses are displayed in Table 2. Our primary outcome is the difference between the average change in SNOT-22 scores from baseline to 6 months between the ESS and control groups. If the average baseline scores happen to be different between the two groups, comparison of the follow-up scores or change scores will lead to under- or overestimation of the treatment effect. Therefore, the analysis of covariance will be used where possible baseline imbalances are controlled for. The estimate for difference between the means will be calculated with 95% confidence intervals. Statistical significance will not be presented for secondary outcomes to avoid multicomparison problem.

**Table 2:** Variables, measures, and planned statistical analyses. ESS, endoscopic sinus surgery; SNOT-22, Sinonasal Outcome Test Quality of Life Questionnaire; RAND 36, Research and Development 36 Item Health Survey; MIC, minimal clinically important change; CI, confidence interval; NNT, number needed to treat

Variable/outcome	Hypothesis	Outcome measure	Method of analysis
Primary outcome	ESS improves outcome from baseline to 6 months	Difference between the mean SNOT-22 change scores of ESS and control group	Analysis of covariance, estimate for difference between means with 95% CI (adjusted age (≤ 50 vs. > 50 years), sex, and allergy status (yes vs. no))
			Distributions of different SNOT-22 domain scores reported in ESS and control group
Secondary outcomes			
General QoL change	Improvement occurs	Difference between the mean RAND-36 change scores of ESS and control group	Analysis of covariance
Proportion benefiting	Improvement occurs	Difference in proportions benefiting (SNOT-22 > MIC) in ESS and control group	Chi-squared test, risk ratio with 95% CI, NNT
No. of episodes, visits, antibiotic courses, sick days	Improvement occurs	Difference in number of episodes, visits, antibiotic courses, and sick days in ESS and control group	Difference in means with 95% CI
No. of symptomatic days	Improvement occurs	Difference in no. of days with nasal obstruction, nasal discharge, facial pain/pressure, and fever in ESS and control group	Difference in means with 95% CI
No. of postoperative complications	Improvement occurs	Frequency of postoperative synechia formations, infections, nasal pain, bleeding, and orbital and intracranial complications	Number (%)

### Interim analyses {21b}

No interim analyses will be done.

### Methods for additional analyses (e.g., subgroup and adjusted analyses) {20b}

We plan to conduct two subgroup analyses. We know that both age and sex will probably affect quality of life and medical care seeking. Firstly, we will compare the estimates for difference between the mean SNOT-22 scores between the ESS and control groups both in males and females. Secondly, we will do the same analysis in participants aged ≤ 50 and > 50 years of age. We anticipate that the treatment effect will be larger among both females and younger participants.

If there is imbalance in baseline variables (age, sex, allergy status) between groups, we also perform adjusted analyses by including the following baseline variables in the analysis of covariance: age (≤ 50 vs. > 50 years), sex, and allergy status (yes vs. no).

### Methods in analysis to handle protocol non-adherence and any statistical methods to handle missing data {20c}

An “as randomized” analysis is performed which retains participants in the group to which they were originally allocated (intention to treat principle). Outcome data obtained from all participants are included in the data analysis, regardless of protocol adherence. Per protocol analysis will be performed as sensitivity analysis. In case there is missing data on the primary outcome, a multiple imputation method will be used.

### Plans to give access to the full protocol, participant level-data and statistical code {31c}

The data collected will be kept in a secure cabinet. Only the research members will have access to the files. After the completion of the study, the results will be made public through publication in a scientific journal along with conferences related to ear, nose, and throat, as well as the ClinicalTrials.gov website. The data generated or analyzed during this study will be available from the corresponding author on reasonable request. The protocol will be sent to a journal for publication.

## Oversight and monitoring

### Composition of the coordinating center and trial steering committee {5d}

The research group is responsible for participant safety, study design, database integrity, and study conduct. The group as a whole and particularly its leader (O.-P.A.) and statistician (P.O.) have long experience in observational and interventional clinical trials. The group has wide clinical experience on the medical condition being studied. The group deals with any clinical or scientific problems together. The group leader is primarily responsible for the ethnical aspects of this project including data management and storage. The Oulu University Hospital’s administrative leader has granted a permission to perform this research in the hospital.

### Composition of the data monitoring committee, its role and reporting structure {21a}

This trial includes only conventional treatments and thus trial participation specifically exposes to no extra risk of any complication. Therefore, this project has no data monitoring committee.

### Adverse event reporting and harms {22}

ESS operation includes risks of postoperative bleeding, postoperative infections, pain and intranasal synechia formation, and uncommon risks of cerebrospinal fluid leak or orbital injury. The study participants will be recruited from a group of patients that are usually operated on our clinics without a study setting, so the study itself does not add any risk for participants as there is no difference in surgical methods. In case of complications, participants are instructed to contact their respective clinic and their care is arranged by the study hospitals according to good clinical practice. All study personnel are employees of the trial hospital and will be insured by their employer.

We collect data about potential harms and will report our findings. The study logbook includes entries for nasal pain, facial pain, and bleeding. Post-operational scarring is assessed with endoscopies during follow-up. Severe harms are recorded from patient files.

The study includes either CBCT or CT scan of the sinuses. Preoperative imaging is done routinely in our hospital for every ESS candidate both to explore the extent of the sinonasal disease and to provide a chart for the possible ESS operation to enhance safety. A CT scan causes radiation exposure equal to about 1.5 months and a CBCT about 7 days of background radiation. All participants will be informed of this.

### Frequency and plans for auditing trial conduct {23}

External auditing is not planned. The study subjects receive standard care in accordance with rhinosinusitis guidelines.

### Plans for communicating important protocol amendments to relevant parties {25}

All research group members may introduce protocol amendments. These are considered then together, and the principal investigator will be responsible for the final decision to amend and how the substantive changes are communicated to relevant stakeholders (The Northern Ostrobothnia Hospital District’s ethical committee and ClinicalTrials.gov register). The protocol version with a date and list of amendments is clearly presented in the protocol. There are no amendments so far.

### Dissemination plans {31a}

After the completion of the study, the results will be made public through publication in a scientific journal, at conferences related to ear, nose, and throat diseases, and on the ClinicalTrials.gov website.

## Discussion

This protocol deals with an open label randomized controlled trial that explores the quality of life change 6 months after endoscopic sinus surgery (ESS) versus conventional conservative treatment among adult patients suffering from recurrent acute rhinosinusitis episodes (RARS). Observational studies have presented information that, in RARS patients, ESS results in diminished symptoms, numbers of medical visits, and days lost from work or studies and enhanced quality of life [2–6, 10, 11]. Moreover, these benefits have been gained without serious adverse events. However, to our knowledge, the effect of ESS has not been investigated in a randomized controlled trial. Because of this, the role of ESS in the treatment of RARS has been somewhat vague in international guidelines. The trial we have started will give a more accurate estimate of the effects of ESS on quality of life and on various objective parameters reflecting the rhinosinusitis infections among adult RARS patients.

A rhinosinusitis episode starts as a viral infection, which naturally cannot be influenced by any surgical therapy. Some of the episodes are complicated by a bacterial infection of the sinuses, though [28]. Here, the surgical opening of particularly the ethmoidal and maxillary sinuses may improve drainage and clearance of these sinuses. This in turn may prevent and shorten the symptoms leading to fewer, milder, and shorter rhinosinusitis episodes and thus improved quality of life.

There are some strengths and limitations regarding our study. As practically all ESS procedures in the area are done in our hospital, the participant sample is population-based. Moreover, we only include primary surgery, as cases undergoing revision surgery are more complex. We use block randomization to ensure that the follow-up times (calendar times) of participants will be evenly distributed in both groups. A limitation is that this is a single-center study. A multicenter study would be helpful in providing data that are more significant and would increase the generalizability of the findings. The fact that this is an open label trial forms another limitation. Knowledge of the intervention may cause ascertainment bias in the measurement of outcomes, performance bias in the decision to discontinue or modify interventions, or other aspects of care, and exclusion/attrition bias in the decision to withdraw from the trial. The wait-time for ESS is restricted by law to no more than 6 months, which resulted in a relatively short follow-up. However, we think that the short-term effect of ESS shows its overall usefulness. Earlier research has shown that the objective outcomes after ESS do not depend on the length of follow-up [29].

## Trial status

Recruitment began 4 February 2020 and is currently ongoing. We anticipate recruitment to end in 2024, although recruitment was momentarily stalled in the spring of 2020 because of the SARS-CoV-2 pandemic. Final version of the protocol is version number seven, dated 25 August 2021.

## Supplementary Information

**Additional file 1.** Medical History. The baseline questionnaire which the study participants fill manually.

**Additional file 2.** Study Logbook. The translated version of the logbook given to participants to be filled manually during the follow-up.

**Additional file 3.** Lund-Mackay stage and Lund-Kennedy score forms. Radiological and endoscopical assessment forms that an attending member of the research team fills during hospital visits.

## Data Availability

The data will not be publicly available (no urn reference). However, it will be available from the corresponding author on reasonable request.

## Abbreviations

CBCT: Cone beam computed tomography
CT: Computed tomography
ESS: Endoscopic sinus surgery
QoL: Quality of life
RAND-36: Research and Development 36-item Health Survey
RARS: Recurrent acute rhinosinusitis
SD: Standard deviation
SNOT-22: Sinonasal Outcome Test 22
