# Supplementary material for: Endoscopic sinus surgery (ESS) to change quality of life for adults with recurrent rhinosinusitis: study protocol for a randomized controlled trial
Source: Trials. 2021 Sep 8;22:606. doi: 10.1186/s13063-021-05576-z (PMC8424164; doi:10.1186/s13063-021-05576-z)
Supplement: Supplementary file 1 — Additional file 1. Medical History. The baseline questionnaire which the study participants fill manually. [file 13063_2021_5576_MOESM1_ESM.docx]

**Medical History**

**1.** Name: _______________________________________

**2.** Social security number: _______________________________________

**3.** Date __________

**4.** Age: _____ years **5.** Gender: 1) male 2) female

**6.** Smoking _________years **7.** Smoking __________ cigarettes per day

**8.** Currently smoking

**9.** Height ______cm **10.** Weight _______kg

Education (please circle the right option) **11**. Primary school

**11**. Comprehensive school

**11**. High school, polytechnic, or university

Which of the following do You have? (please circle)

**12.** asthma

**13.** nasal polyps (that a doctor has removed before)

**14.** aspirin sensitivity (rash or mucosal reaction from aspirin)

**15**. other respiratory disease

**16**. nasal septum operated on before

**17**. nasal sinuses operated on before

**18.** radiation therapy given to the nasal area

**19.** nasal tumour

**20.** medication for hypertension (beta blocker)

**21**. combined oral contraceptives

**22**. previous nasal trauma requiring medical treatment (e.g. broken nose)

**23**. reflux that required medical treatment

**24**. compromised immune system (e.g. medication for rheumatism, antibody deficiency, HIV)

**25**. diabetes

**26.** thyroid hypofunction

**27**. systemic disease (sarcoidosis, lupus, Wegener’s granulomatosis, vasculitis)

**28**. dental infection

**29.** cystic fibrosis

**30.** ciliary dysfuction

**31**. currently pregnant

**32.** Do You have any long-term disease, defect or disability? (all long-term conditions diagnosed by a physician and any condition that has lasted at least 3 months not diagnosed by a physician but affecting working ability or daily performance)

YES NO

**33.** If You answered yes above, does the condition hinder Your ability to work or execute Your daily activities? (circle the correct option)

a lot somewhat a little not at all

Do You have allergic rhinitis symptoms caused by (circle all the correct options)?

**34.** pollen

**35.** animal dandruff

**36**. dust

**37.** mold

**38**. I’m allergic but I don’t know what to.

**39**. My close relatives have asthma, allergic rhinitis or nasal polyps.

Do You have any of the following? (circle all the correct options)

**40**. Domestic animal (cat, dog, horse etc.)

**41.** suspected bad indoor air quality at home

**42**. suspected bad indoor air quality at work

**43.** hobbies exposing to irritating substances (gases, paints etc.)

**44**. suspected occupational disease – baker

**45**. suspected occupational disease – farmer (cattle)

**46**. suspected occupational disease – other

**47.** Have You had any sinus infection during the last 2 years? (please circle the correct option)

YES NO

**48.** If You answered yes above, how many episodes have You had per year? (please give the answer in numbers)

_________

How was the diagnosis of a sinus infection made? (please circle all the correct options)

**49**. by looking into the nose

**50**. with an ultrasound examination

**51**. with an x-ray

**52**. with a maxillary puncture

**53.** Have You had a sinus infection during the past month? (please circle the correct option)

YES NO

**54**. Have You had nasal congestion during the past month? (please circle the correct option)

YES NO

**55.** Do You currently have nasal congestion? (please circle the correct option)

YES NO

During the past month, have You used some of the following medications? (please circle the correct options)

**56**. I have not.

**57**. Nasal decongestant (e.g. Nasolin, Otrivin, Naso-ratiopharm)

**58**. Nasal steroid (e.g. Beclonase, Nasacort, Nasonex, Flixonase, Rhinocort, Avamys)

**59.** Antihistamine (e.g. Aerius, Xyzal, Heinix, Semprex)

**60**. Combination medicine (e.g. Duact, Clarinase, Cirrus, Aerinaze)

**61**. Anticholinergic medicine (Atrovent Nasal)

**62**. Antileukotriene medicine (Singulair, Accolate)

**63**. Other, what ___________________________

**64.** Have You used nasal decongestants for over 6 months continuously? (please circle the correct option)

YES NO

**65.** Have You used oral prednisolone during the past month? (please circle the correct option)

YES NO

**66.** If You are receiving allergen desensitisation for Your allergy, how many months of treatment have You received?

_______ months

**67.** Have You previously received allergen desensitisation for Your allergy? (please circle the correct option)

YES NO

**68.** Have You used some method of nasal moistening during the past month (e.g. nasal douching, Humidose, A-Vita nasal drops, Nozoil)? (please circle the correct option)

YES NO

**69.** How much have the medications You have used improved Your nasal congestion? (mark on the line)

├─────────────────────────────┤

not at all completely (no symptoms)

**70.** How many times have You visited a physician during the past 6 months? (please circle the correct option)

no visits 1-2 visits 3 or more visits
