# Supplementary material for: Endoscopic sinus surgery (ESS) to change quality of life for adults with recurrent rhinosinusitis: study protocol for a randomized controlled trial
Source: Trials. 2021 Sep 8;22:606. doi: 10.1186/s13063-021-05576-z (PMC8424164; doi:10.1186/s13063-021-05576-z)
Supplement: Supplementary file 2 — Additional file 2. Study Logbook. The translated version of the logbook given to participants to be filled manually during the follow-up. [file 13063_2021_5576_MOESM2_ESM.docx]

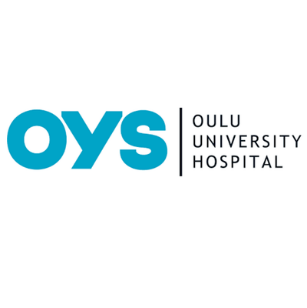


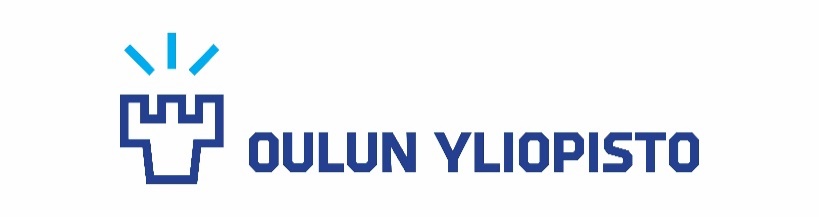


Sinus Surgery in Recurrent Sinus Infections

LOGBOOK

Translated from version 004, 02.12.2019

## Dear participant,

Thank You for Your willingness to take part in our study. Recurrent sinus infections cause many medical visits, courses of medication and absences from work or school as well as wearisome symptoms. Previous studies indicate that endoscopic sinus surgery diminishes symptoms and increases quality of life. Endoscopic sinus surgeries are one of the most common operations in the field of ear, nose and throat diseases. Thus, we think that studying the surgical treatment of sinus infections is important.

Our current study aims at investigating how endoscopic sinus surgery affects patients’ quality of life, occurrence of symptoms and number of sinus infections. A large group of patients suffering from sinus infections will participate in this study.

This logbook includes a symptom diary that we ask You to fill in during Your time in the study. The logbook also contains a form for possible doctor’s visits and questionnaires about Your symptoms and the surgical procedure. Some of these are to be filled in before and after Your sinus operation. You will be separately informed of the study processes.

We ask You to fill in the symptom diary as precisely as You can in order to gather as high-quality data as possible for this study. It will help us to treat patients suffering from sinus infections as well as we can in the future, too.

Anchor questions filled before sinus surgery

**1. How much do symptoms from Your sinuses disturb You and Your daily life?**

1=not at all 2=very little 3=a little 4= somewhat 5=quite a bit 6= a lot 7= tremendously

**2. With Your current knowledge and experience, would You recommend sinus surgery to a friend?**

1=absolutely not
2=probably not
3=maybe
4=probably
5=absolutely

**3. There is a list of harms related to sinus infections below. Which one’s improvement do You think is the most important? Only mark those options that You wish to get relief to.** Mark things in order of importance 1, 2, 3… with 1 being the most important (You do not have to use all numbers).

- Nasal congestion
- Nasal discharge
- Other sinus symptoms (pressure, pain, loss of the sense of smell)
- Feeling of malady, lack of mental and physical capacity
- Recurring medical visits and courses of medicine
- Absences from work or study
- Social harm or depression

**4. How much should the thing or symptom You marked as the most important (1) improve that You would agree to surgical treatment?**

1=not at all 2=very little 3= little 4= somewhat 5=quite a bit 6= a lot 7= tremendously

| 31 |  |  |  |  |  |  |  |
| --- | --- | --- | --- | --- | --- | --- | --- |
| 30 |  |  |  |  |  |  |  |
| 29 |  |  |  |  |  |  |  |
| 28 |  |  |  |  |  |  |  |
| 27 |  |  |  |  |  |  |  |
| 26 |  |  |  |  |  |  |  |
| 25 |  |  |  |  |  |  |  |
| 24 |  |  |  |  |  |  |  |
| 23 |  |  |  |  |  |  |  |
| 22 |  |  |  |  |  |  |  |
| 21 |  |  |  |  |  |  |  |
| 20 |  |  |  |  |  |  |  |
| 19 |  |  |  |  |  |  |  |
| 18 |  |  |  |  |  |  |  |
| 17 |  |  |  |  |  |  |  |
| 16 |  |  |  |  |  |  |  |
| 15 |  |  |  |  |  |  |  |
| 14 |  |  |  |  |  |  |  |
| 13 |  |  |  |  |  |  |  |
| 12 |  |  |  |  |  |  |  |
| 11 |  |  |  |  |  |  |  |
| 10 |  |  |  |  |  |  |  |
| 9 |  |  |  |  |  |  |  |
| 8 |  |  |  |  |  |  |  |
| 7 |  |  |  |  |  |  |  |
| 6 |  |  |  |  |  |  |  |
| 5 |  |  |  |  |  |  |  |
| 4 |  |  |  |  |  |  |  |
| 3 |  |  |  |  |  |  |  |
| 2 |  |  |  |  |  |  |  |
| 1 |  |  |  |  |  |  |  |
|  | Nasal congestion | Nasal discharge | Facial pain/pressure | Nasal pain | Nasal bleeding | Fever (over 37,5°C) | Absence from work or study |

**Month:___________________ Year:____________**

## Symptom diary

We ask You to mark down Your symptoms in this diary. In the first five rows (nasal congestion, nasal discharge, facial pain or pressure, nasal pain, nasal bleeding), please mark down the number that You feel that best describes the magnitude of the symptom according to the following scale: 1 = mild symptom, 2= moderate symptom, 3 = difficult symptom. Mark the number on the date of the symptom. In the last two rows (fever and absences), please mark down an X on the date of the respective condition.

| 31 |  |  |  |  |  |  |  |
| --- | --- | --- | --- | --- | --- | --- | --- |
| 30 |  |  |  |  |  |  |  |
| 29 |  |  |  |  |  |  |  |
| 28 |  |  |  |  |  |  |  |
| 27 |  |  |  |  |  |  |  |
| 26 |  |  |  |  |  |  |  |
| 25 |  |  |  |  |  |  |  |
| 24 |  |  |  |  |  |  |  |
| 23 |  |  |  |  |  |  |  |
| 22 |  |  |  |  |  |  |  |
| 21 |  |  |  |  |  |  |  |
| 20 |  |  |  |  |  |  |  |
| 19 |  |  |  |  |  |  |  |
| 18 |  |  |  |  |  |  |  |
| 17 |  |  |  |  |  |  |  |
| 16 |  |  |  |  |  |  |  |
| 15 |  |  |  |  |  |  |  |
| 14 |  |  |  |  |  |  |  |
| 13 |  |  |  |  |  |  |  |
| 12 |  |  |  |  |  |  |  |
| 11 |  |  |  |  |  |  |  |
| 10 |  |  |  |  |  |  |  |
| 9 |  |  |  |  |  |  |  |
| 8 |  |  |  |  |  |  |  |
| 7 |  |  |  |  |  |  |  |
| 6 |  |  |  |  |  |  |  |
| 5 |  |  |  |  |  |  |  |
| 4 |  |  |  |  |  |  |  |
| 3 |  |  |  |  |  |  |  |
| 2 |  |  |  |  |  |  |  |
| 1 |  |  |  |  |  |  |  |
|  | Nasal congestion | Nasal discharge | Facial pain/pressure | Nasal pain | Nasal bleeding | Fever (over 37,5°C) | Absence from work or study |

| 31 |  |  |  |  |  |  |  |
| --- | --- | --- | --- | --- | --- | --- | --- |
| 30 |  |  |  |  |  |  |  |
| 29 |  |  |  |  |  |  |  |
| 28 |  |  |  |  |  |  |  |
| 27 |  |  |  |  |  |  |  |
| 26 |  |  |  |  |  |  |  |
| 25 |  |  |  |  |  |  |  |
| 24 |  |  |  |  |  |  |  |
| 23 |  |  |  |  |  |  |  |
| 22 |  |  |  |  |  |  |  |
| 21 |  |  |  |  |  |  |  |
| 20 |  |  |  |  |  |  |  |
| 19 |  |  |  |  |  |  |  |
| 18 |  |  |  |  |  |  |  |
| 17 |  |  |  |  |  |  |  |
| 16 |  |  |  |  |  |  |  |
| 15 |  |  |  |  |  |  |  |
| 14 |  |  |  |  |  |  |  |
| 13 |  |  |  |  |  |  |  |
| 12 |  |  |  |  |  |  |  |
| 11 |  |  |  |  |  |  |  |
| 10 |  |  |  |  |  |  |  |
| 9 |  |  |  |  |  |  |  |
| 8 |  |  |  |  |  |  |  |
| 7 |  |  |  |  |  |  |  |
| 6 |  |  |  |  |  |  |  |
| 5 |  |  |  |  |  |  |  |
| 4 |  |  |  |  |  |  |  |
| 3 |  |  |  |  |  |  |  |
| 2 |  |  |  |  |  |  |  |
| 1 |  |  |  |  |  |  |  |
|  | Nasal congestion | Nasal discharge | Facial pain/pressure | Nasal pain | Nasal bleeding | Fever (over 37,5°C) | Absence from work or study |

**Month:___________________ Year:____________**

**Month:___________________ Year:____________**

| 31 |  |  |  |  |  |  |  |
| --- | --- | --- | --- | --- | --- | --- | --- |
| 30 |  |  |  |  |  |  |  |
| 29 |  |  |  |  |  |  |  |
| 28 |  |  |  |  |  |  |  |
| 27 |  |  |  |  |  |  |  |
| 26 |  |  |  |  |  |  |  |
| 25 |  |  |  |  |  |  |  |
| 24 |  |  |  |  |  |  |  |
| 23 |  |  |  |  |  |  |  |
| 22 |  |  |  |  |  |  |  |
| 21 |  |  |  |  |  |  |  |
| 20 |  |  |  |  |  |  |  |
| 19 |  |  |  |  |  |  |  |
| 18 |  |  |  |  |  |  |  |
| 17 |  |  |  |  |  |  |  |
| 16 |  |  |  |  |  |  |  |
| 15 |  |  |  |  |  |  |  |
| 14 |  |  |  |  |  |  |  |
| 13 |  |  |  |  |  |  |  |
| 12 |  |  |  |  |  |  |  |
| 11 |  |  |  |  |  |  |  |
| 10 |  |  |  |  |  |  |  |
| 9 |  |  |  |  |  |  |  |
| 8 |  |  |  |  |  |  |  |
| 7 |  |  |  |  |  |  |  |
| 6 |  |  |  |  |  |  |  |
| 5 |  |  |  |  |  |  |  |
| 4 |  |  |  |  |  |  |  |
| 3 |  |  |  |  |  |  |  |
| 2 |  |  |  |  |  |  |  |
| 1 |  |  |  |  |  |  |  |
|  | Nasal congestion | Nasal discharge | Facial pain/pressure | Nasal pain | Nasal bleeding | Fever (over 37,5°C) | Absence from work or study |

| 31 |  |  |  |  |  |  |  |
| --- | --- | --- | --- | --- | --- | --- | --- |
| 30 |  |  |  |  |  |  |  |
| 29 |  |  |  |  |  |  |  |
| 28 |  |  |  |  |  |  |  |
| 27 |  |  |  |  |  |  |  |
| 26 |  |  |  |  |  |  |  |
| 25 |  |  |  |  |  |  |  |
| 24 |  |  |  |  |  |  |  |
| 23 |  |  |  |  |  |  |  |
| 22 |  |  |  |  |  |  |  |
| 21 |  |  |  |  |  |  |  |
| 20 |  |  |  |  |  |  |  |
| 19 |  |  |  |  |  |  |  |
| 18 |  |  |  |  |  |  |  |
| 17 |  |  |  |  |  |  |  |
| 16 |  |  |  |  |  |  |  |
| 15 |  |  |  |  |  |  |  |
| 14 |  |  |  |  |  |  |  |
| 13 |  |  |  |  |  |  |  |
| 12 |  |  |  |  |  |  |  |
| 11 |  |  |  |  |  |  |  |
| 10 |  |  |  |  |  |  |  |
| 9 |  |  |  |  |  |  |  |
| 8 |  |  |  |  |  |  |  |
| 7 |  |  |  |  |  |  |  |
| 6 |  |  |  |  |  |  |  |
| 5 |  |  |  |  |  |  |  |
| 4 |  |  |  |  |  |  |  |
| 3 |  |  |  |  |  |  |  |
| 2 |  |  |  |  |  |  |  |
| 1 |  |  |  |  |  |  |  |
|  | Nasal congestion | Nasal discharge | Facial pain/pressure | Nasal pain | Nasal bleeding | Fever (over 37,5°C) | Absence from work or study |

**Month:___________________ Year:____________**

**Month:___________________ Year:____________**

| 31 |  |  |  |  |  |  |  |
| --- | --- | --- | --- | --- | --- | --- | --- |
| 30 |  |  |  |  |  |  |  |
| 29 |  |  |  |  |  |  |  |
| 28 |  |  |  |  |  |  |  |
| 27 |  |  |  |  |  |  |  |
| 26 |  |  |  |  |  |  |  |
| 25 |  |  |  |  |  |  |  |
| 24 |  |  |  |  |  |  |  |
| 23 |  |  |  |  |  |  |  |
| 22 |  |  |  |  |  |  |  |
| 21 |  |  |  |  |  |  |  |
| 20 |  |  |  |  |  |  |  |
| 19 |  |  |  |  |  |  |  |
| 18 |  |  |  |  |  |  |  |
| 17 |  |  |  |  |  |  |  |
| 16 |  |  |  |  |  |  |  |
| 15 |  |  |  |  |  |  |  |
| 14 |  |  |  |  |  |  |  |
| 13 |  |  |  |  |  |  |  |
| 12 |  |  |  |  |  |  |  |
| 11 |  |  |  |  |  |  |  |
| 10 |  |  |  |  |  |  |  |
| 9 |  |  |  |  |  |  |  |
| 8 |  |  |  |  |  |  |  |
| 7 |  |  |  |  |  |  |  |
| 6 |  |  |  |  |  |  |  |
| 5 |  |  |  |  |  |  |  |
| 4 |  |  |  |  |  |  |  |
| 3 |  |  |  |  |  |  |  |
| 2 |  |  |  |  |  |  |  |
| 1 |  |  |  |  |  |  |  |
|  | Nasal congestion | Nasal discharge | Facial pain/pressure | Nasal pain | Nasal bleeding | Fever (over 37,5°C) | Absence from work or study |

| 31 |  |  |  |  |  |  |  |
| --- | --- | --- | --- | --- | --- | --- | --- |
| 30 |  |  |  |  |  |  |  |
| 29 |  |  |  |  |  |  |  |
| 28 |  |  |  |  |  |  |  |
| 27 |  |  |  |  |  |  |  |
| 26 |  |  |  |  |  |  |  |
| 25 |  |  |  |  |  |  |  |
| 24 |  |  |  |  |  |  |  |
| 23 |  |  |  |  |  |  |  |
| 22 |  |  |  |  |  |  |  |
| 21 |  |  |  |  |  |  |  |
| 20 |  |  |  |  |  |  |  |
| 19 |  |  |  |  |  |  |  |
| 18 |  |  |  |  |  |  |  |
| 17 |  |  |  |  |  |  |  |
| 16 |  |  |  |  |  |  |  |
| 15 |  |  |  |  |  |  |  |
| 14 |  |  |  |  |  |  |  |
| 13 |  |  |  |  |  |  |  |
| 12 |  |  |  |  |  |  |  |
| 11 |  |  |  |  |  |  |  |
| 10 |  |  |  |  |  |  |  |
| 9 |  |  |  |  |  |  |  |
| 8 |  |  |  |  |  |  |  |
| 7 |  |  |  |  |  |  |  |
| 6 |  |  |  |  |  |  |  |
| 5 |  |  |  |  |  |  |  |
| 4 |  |  |  |  |  |  |  |
| 3 |  |  |  |  |  |  |  |
| 2 |  |  |  |  |  |  |  |
| 1 |  |  |  |  |  |  |  |
|  | Nasal congestion | Nasal discharge | Facial pain/pressure | Nasal pain | Nasal bleeding | Fever (over 37,5°C) | Absence from work or study |

**Month:___________________ Year:____________**

**Month:___________________ Year:____________**

## Doctor’s visits

Please mark down the date, place You visited, diagnosis or symptom and possible treatment assigned for You.

Date­­­­­­­­­­­__________________ Place_____________________________
Diagnosis/symptom____________________________ Treatment ___________________________________

Date­­­­­­­­­­­__________________ Place_____________________________
Diagnosis/symptom____________________________ Treatment ___________________________________

Date­­­­­­­­­­­__________________ Place_____________________________
Diagnosis/symptom____________________________ Treatment ___________________________________

Date­­­­­­­­­­­__________________ Place_____________________________
Diagnosis/symptom____________________________ Treatment ___________________________________

Date­­­­­­­­­­­__________________ Place_____________________________
Diagnosis/symptom____________________________ Treatment ___________________________________

Date­­­­­­­­­­­__________________ Place_____________________________
Diagnosis/symptom____________________________ Treatment ___________________________________

Date­­­­­­­­­­­__________________ Place_____________________________
Diagnosis/symptom____________________________ Treatment ___________________________________

Date­­­­­­­­­­­__________________ Place_____________________________
Diagnosis/symptom____________________________ Treatment ___________________________________

Date­­­­­­­­­­­__________________ Place_____________________________
Diagnosis/symptom____________________________ Treatment ___________________________________

Date­­­­­­­­­­­__________________ Place_____________________________
Diagnosis/symptom____________________________ Treatment ___________________________________

Date­­­­­­­­­­­__________________ Place_____________________________
Diagnosis/symptom____________________________ Treatment ___________________________________

Date­­­­­­­­­­­__________________ Place_____________________________
Diagnosis/symptom____________________________ Treatment ___________________________________

Date­­­­­­­­­­­__________________ Place_____________________________
Diagnosis/symptom____________________________ Treatment ___________________________________

Date­­­­­­­­­­­__________________ Place_____________________________
Diagnosis/symptom____________________________ Treatment ___________________________________

Date­­­­­­­­­­­__________________ Place_____________________________
Diagnosis/symptom____________________________ Treatment ___________________________________

Date­­­­­­­­­­­__________________ Place_____________________________
Diagnosis/symptom____________________________ Treatment ___________________________________

Date­­­­­­­­­­­__________________ Place_____________________________
Diagnosis/symptom____________________________ Treatment ___________________________________

Date­­­­­­­­­­­__________________ Place_____________________________
Diagnosis/symptom____________________________ Treatment ___________________________________

Date­­­­­­­­­­­__________________ Place_____________________________
Diagnosis/symptom____________________________ Treatment ___________________________________

Date­­­­­­­­­­­__________________ Place_____________________________
Diagnosis/symptom____________________________ Treatment ___________________________________

Date­­­­­­­­­­­__________________ Place_____________________________
Diagnosis/symptom____________________________ Treatment ___________________________________

Date­­­­­­­­­­­__________________ Place_____________________________
Diagnosis/symptom____________________________ Treatment ___________________________________

Date­­­­­­­­­­­__________________ Place_____________________________
Diagnosis/symptom____________________________ Treatment ___________________________________

Date­­­­­­­­­­­__________________ Place_____________________________
Diagnosis/symptom____________________________ Treatment ___________________________________

Date­­­­­­­­­­­__________________ Place_____________________________
Diagnosis/symptom____________________________ Treatment ___________________________________

Date­­­­­­­­­­­__________________ Place_____________________________
Diagnosis/symptom____________________________ Treatment ___________________________________

Date­­­­­­­­­­­__________________ Place_____________________________
Diagnosis/symptom____________________________ Treatment ___________________________________

Date­­­­­­­­­­­__________________ Place_____________________________
Diagnosis/symptom____________________________ Treatment ___________________________________

## Patient instruction in case of falling ill

During Your participation in the study keep record of Your symptoms. If You feel like You need to consult a doctor during Your participation, please act as follows:

- If You become ill during Your time in the study and need a doctor, we ask You to contact Your ear, nose and throat disease clinic in Oulu University Hospital during weekdays. During holidays and weekends, please contact Your local health center or another doctor as You choose.
- If You otherwise have upper respiratory symptoms or other ailments, please contact Your local health center or other doctor as You choose.
- If You are hesitant of whether to consults a doctor, contact us with a phone call.
  - Oulu University Hospital, Ear, nose and throat disease clinic phone no. 08 315 3494. Contact period Mon-Fri 8:30-10:30 and 13:00-14:00.

## Dear doctor,

This patient is participating in a study that investigates the effect of sinus surgery on recurrent acute rhinosinusitis. If You diagnose an acute rhinosinusitis with this patient, please act as follows:

- Examine the patient as you usually would and mark the findings in the medical records.
- Start treatment with antibiotics if You would start it without this patient being in a study.

## Anchor questions filled in after sinus surgery

**1. 1. How much do symptoms from Your sinuses disturb You and Your daily life?**

1=not at all 2=very little 3=a little 4= somewhat 5=quite a bit 6= a lot 7= tremendously

**2. With Your current knowledge and experience, would You recommend sinus surgery to a friend?**

1=absolutely not
2=probably not
3=maybe
4=probably
5=absolutely

**3. There is a list of harms related to sinus infections below. Which one’s improvement after surgery do You think is the most important? Only mark those options that You’ve gotten relief to.** Mark things in order of importance 1, 2, 3… with 1 being the most important (You do not have to use all numbers).

- Nasal congestion
- Nasal discharge
- Other sinus symptoms (pressure, pain, loss of the sense of smell)
- Feeling of malady, lack of mental and physical capacity
- Recurring medical visits and courses of medicine
- Absences from work or study
- Social harm or depression

**4. How much should the thing or symptom You marked as the most important (1) improve that You would agree to surgical treatment?**

1=not at all 2=very little 3=a little 4= somewhat 5=quite a bit 6= a lot 7= tremendously

**5. Have You had symptoms after the surgery?**

1 = much more 2 = somewhat more 3 = the same amount 4 = somewhat less 5 = much less

Further information when needed from:

Heidi Kaski, MD

Ear, nose and throat disease clinic, 90220 Oulu University Hospital

heidi.m.kaski@gmail.com

Or Antti Alakärppä, MD, PhD, specialist

Ear, nose and throat disease clinic, 90220 Oulu University Hospital

phone no. 08 315 3471

­­­­­­­­­­­­­________________________________________________________________________________

Thank you for Your co-operation

Heidi Kaski, MD
