# Supplementary material for: Endoscopic sinus surgery (ESS) to change quality of life for adults with recurrent rhinosinusitis: study protocol for a randomized controlled trial
Source: Trials. 2021 Sep 8;22:606. doi: 10.1186/s13063-021-05576-z (PMC8424164; doi:10.1186/s13063-021-05576-z)
Supplement: Supplementary file 3 — Additional file 3. Lund-Mackay stage and Lund-Kennedy score forms. Radiological and endoscopical assessment forms that an attending member of the research team fills during hospital visits. [file 13063_2021_5576_MOESM3_ESM.docx]

# Name: Social security number:

# Nasal Endoscopy Lund-Kennedy Score

|  | RIGHT | LEFT |
| --- | --- | --- |
| POLYPS   - No polyps = 0 - Polyps confined to the middle meatus = 1 - Polyps beyond the middle meatus = 2 |  |  |
| DISCHARGE   - No discharge = 0 - Clear, thin discharge = 1 - Thick, purulent discharge = 2 |  |  |
| EDEMA   - No edema = 0 - Mild edema = 1 - Severe edema = 2 |  |  |
| Total points: |  | /12 p. |

# Computed Tomography Lund-Mackay Stage

| 0 = Normal, 1 = Partial Opacification, 2 = Total Opacification | RIGHT | LEFT |
| --- | --- | --- |
| Maxillary sinus |  |  |
| Anterior ethmoid sinus |  |  |
| Posterior ethmoid sinus |  |  |
| Sphenoid sinus |  |  |
| Frontal sinus |  |  |
| Osteomeatal complex |  |  |
| Total points: |  | /24 p. |
